# Human endometriotic lesion expression of the miR-144-3p/miR-451a cluster, its correlation with markers of cell survival and origin of lesion content

**DOI:** 10.1038/s41598-019-45243-7

**Published:** 2019-06-19

**Authors:** Warren B. Nothnick, Kimberly Swan, Rebecca Flyckt, Tommaso Falcone, Amanda Graham

**Affiliations:** 1 Department of Molecular and Integrative Physiology, University of Kansas Medical Center, Kansas City, KS 66160 USA; 2 Department of Obstetrics and Gynecology, University of Kansas Medical Center, Kansas City, KS 66160 USA; 3 Center for Reproductive Sciences, Institute of Reproductive and Perinatal Research, University of Kansas Medical Center, Kansas City, KS 66160 USA; 4 Department of Obstetrics, Gynecology and Women’s Health Institute, Cleveland Clinic, Cleveland, Ohio 44195 USA

**Keywords:** Translational research, Experimental models of disease

## Abstract

Endometriosis is an inflammatory condition in which endometrial tissue grows in ectopic locations. Survival and growth of these ectopic lesions is associated with pain and infertility. MicroRNAs (miRNAs) have been postulated to play a role in the pathophysiology of the disease and we have previously demonstrated expression of miR-451 in human endometriotic lesion tissue. Here we report elevated expression of the miR-144-3p/miR-451a cluster in human endometriotic lesion tissue. Use of an endometriotic epithelial cell line (12Z) in which the miRNA processing enzyme, DROSHA, was knocked down resulted in an enrichment in the primary (pri) form of miR-144-3p but not that of pri-miR-451a. Using an experimental mouse model of endometriosis in which ectopic endometriotic lesions were deficient for both of these miRNAs revealed that miR-451a, but not miR-144-3p may be derived from exogenous sources such as the circulation/erythrocytes. Together, these data suggest that the miR-144-3p/miR-451a cluster is expressed in human endometriotic lesion tissue, the level of expression correlates with survival status of the lesion tissue and that miR-451a, but not miR-144-3p may be derived from exogenous sources such as erythrocytes.

## Introduction

Endometriosis is a debilitating disease in which endometrial stroma and glands grow in ectopic locations. The disease is characterized by pelvic pain, dysmenorrhea and infertility^1^, as well as having a profound impact on an individual’s psychological and social functioning^2–4^. The most well-supported mechanism by which endometriosis is proposed to develop is via reverse menstruation of viable endometrial tissue into the peritoneal cavity. However, because almost all women of reproductive age exhibit some degree of retrograde menstruation^5,6^, it is postulated that additional factors must contribute to the development and progression of the disease. Potential factors such as alterations in the immune system^7^, tissue remodeling factors^8^, inflammatory mediators^9,10^, stem cells^11^ and altered cell survival/apoptosis^12^ have all been proposed, attesting to the complexity of the disease.

MicroRNAs (miRNAs) have been proposed to play a role in the pathogenesis of endometriosis and have garnered considerable attention within the past decade^13^. miRNAs are small non-coding regulatory RNAs that regulate gene expression post-transcriptionally^14^. These regulatory small RNAs have been implicated to play essential roles in many cellular events which are conducive to endometriosis development such as cellular proliferation, invasion and apoptosis^15^. miRNA expression profiles have been established for endometriosis in both the disease tissue and eutopic endometrium as well as eutopic endometrium from control patients^1,16–20^.

*miRNA-451* (*miR-451*; now referred to as *miR-451a*) is one of the most studied miRNAs in endometriosis pathophysiology. Based upon bioinformatic programs such as TargetScan^21^ and mirDIP^22^ which predict miRNA binding target transcripts, both macrophage migration inhibitory (MIF)^23^ and tyrosine 3-monooxygenase/tryptophan 5-monooxygenase activation protein zeta (YWHAZ)^24^ have been proposed targets of *miR-451a* which have been examined with respect to endometriosis pathophysiology. However, as *miR-451a* is expressed as part of the *miR-144-3p*/*miR-451a* cluster^25^, the expression of both miRNAs products has not been evaluated. This may be important as *miR-144-3p* is predicted to target several factors relevant to the pathophysiology of endometriosis including PTGS2/COX2^26–28^, TNF-α^29,30^, IL-1β^29,30^, and IL-6^30,31^. To define the potential role of *miR-144-3p* in the pathophysiology of endometriosis and evaluate the mechanisms for expression of the *miR-144-3p*/*miR-451a* cluster, we performed the following series of experiments.

## Results

### Pri- and mature miR-144-3p expression in human endometriotic lesion tissue

**Figure 1:**
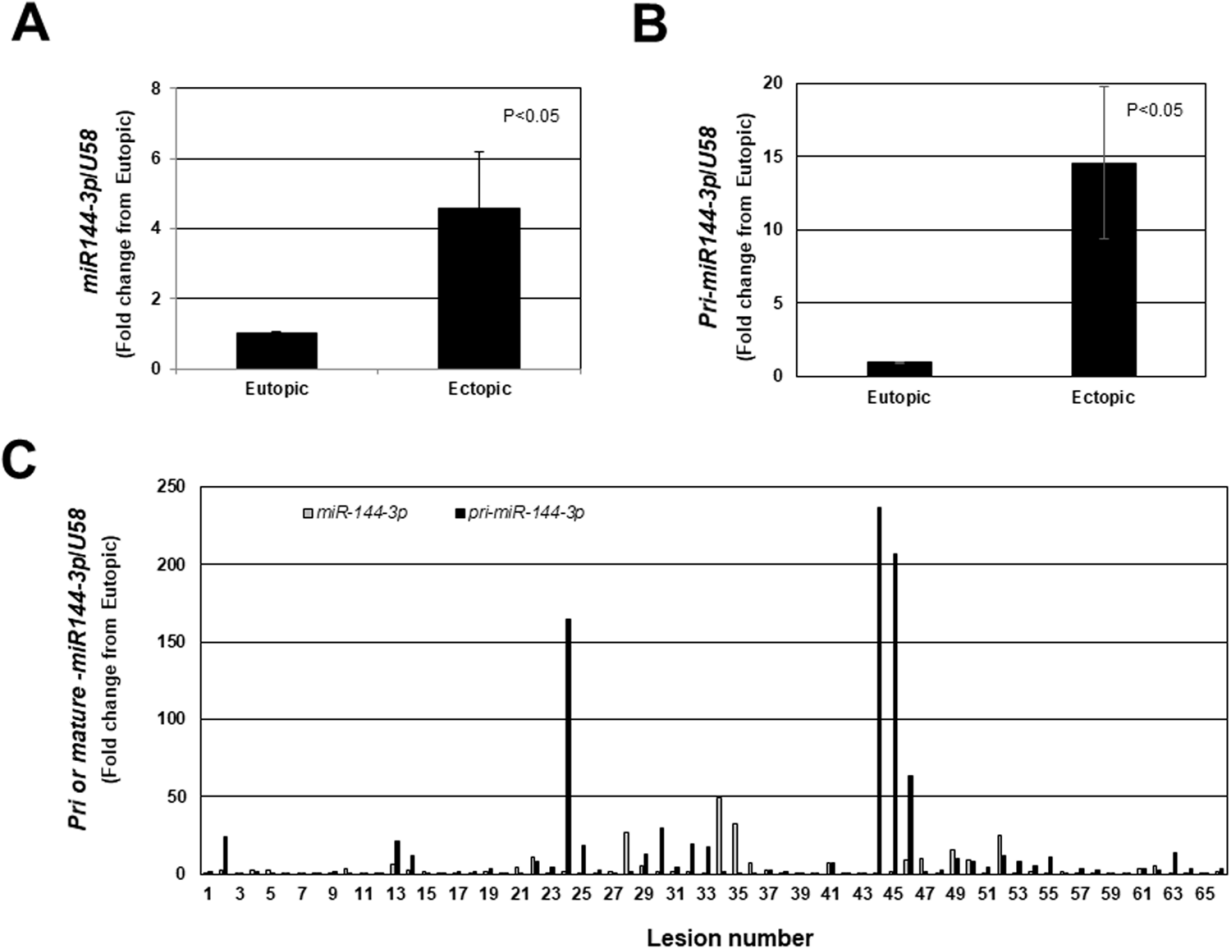
Mature *miR-144-3p* and pri-*miR-144-3p* average and individual lesion expression in human endometriotic lesion tissue. Matched endometriotic lesion and corresponding eutopic endometrial tissue was processed for RNA isolation and *miR-144-3p* (**A**) and *pri-miR-144-3p* (**B**) expression was determined by qRT-PCR as described under “Materials and Methods”. All data are displayed as the mean ± the standard error of the mean (SEM) and p-values are indicated for each assessment for eutopic endometrial samples. (**C**) Ratio (fold change from eutopic tissue expression) of mature *miR-144-3p* and pri-*miR-144-3p* in each individual lesion (N = 66). Data did not pass normality testing and were therefore analyzed using the non-parametric Mann-Whitney test.

**Figure 2:**
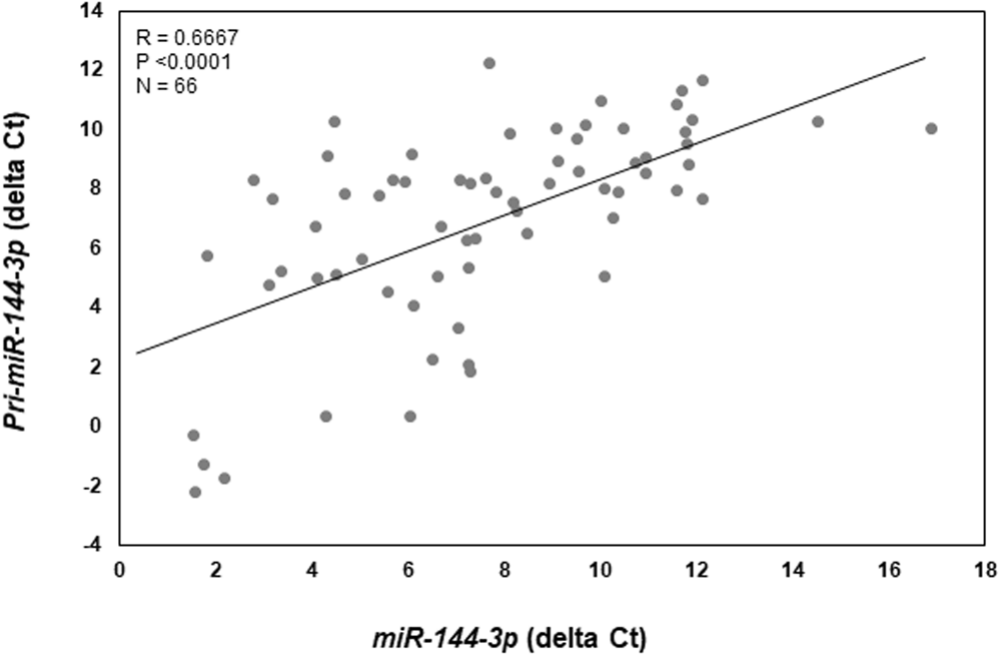
Correlation between mature *miR-144-3p* and pri-*miR-144-3p* in individual endometriotic lesions. Delta ct values (mature *miR-144-3p* or pri-*miR-144-3p* – U58) were plotted and Spearman’s correlation coefficient was calculated. There was a significant positive correlation in lesion expression between mature- and pri-*miR-144-3p*.

Compared to matched eutopic endometrial expression, endometriotic lesion, pri-*miR-144-3p* (Fig. 1A) as well as mature *miR-144-3p* (Fig. 1B) overall expression was significantly greater. Assessment of individual lesion expression of both pri- and mature *miR-144-3p* revealed not only heterogeneity in the level of expression but also the ratio of pri- to mature form (Fig. 1C). Regardless, there was a positive correlation (R = 0.6667; P < 0.0001) between pri-*miR-144-3* and mature *miR-144-3p* in endometriotic lesion tissue (Fig. 2).

**Figure 3:**
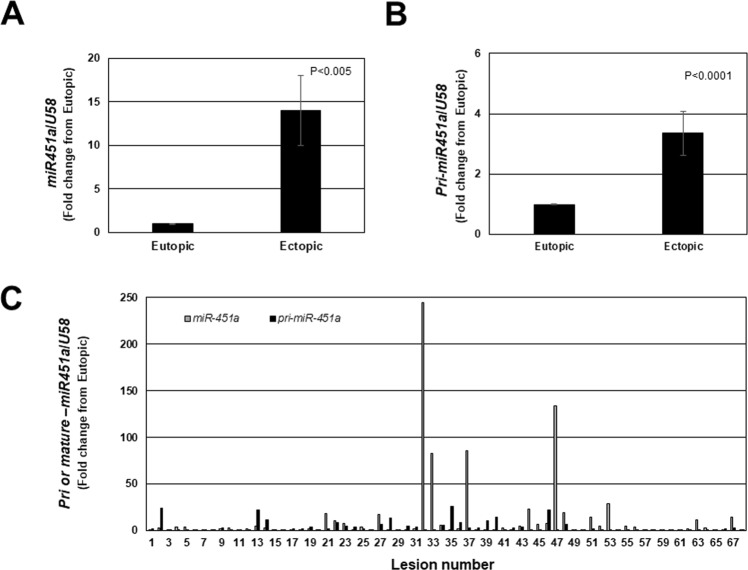
Mature *miR-451a* and pri-*miR-451a* average and individual lesion expression in human endometriotic lesion tissue. Matched endometriotic lesion and corresponding eutopic endometrial tissue was processed for RNA isolation and *miR-451a* (**A**) and *pri-miR-451a* (**B**) expression was determined by qRT-PCR as described under “Materials and Methods”. All data are displayed as the mean ± the standard error of the mean (SEM) and p-values are indicated for each assessment for eutopic endometrial samples. (**C**) Ratio (fold change from eutopic tissue expression) of mature *miR-451a* and pri-*miR-451a* in each individual lesion (N = 68). Data did not pass normality testing and were therefore analyzed using the non-parametric Mann-Whitney test.

**Figure 4:**
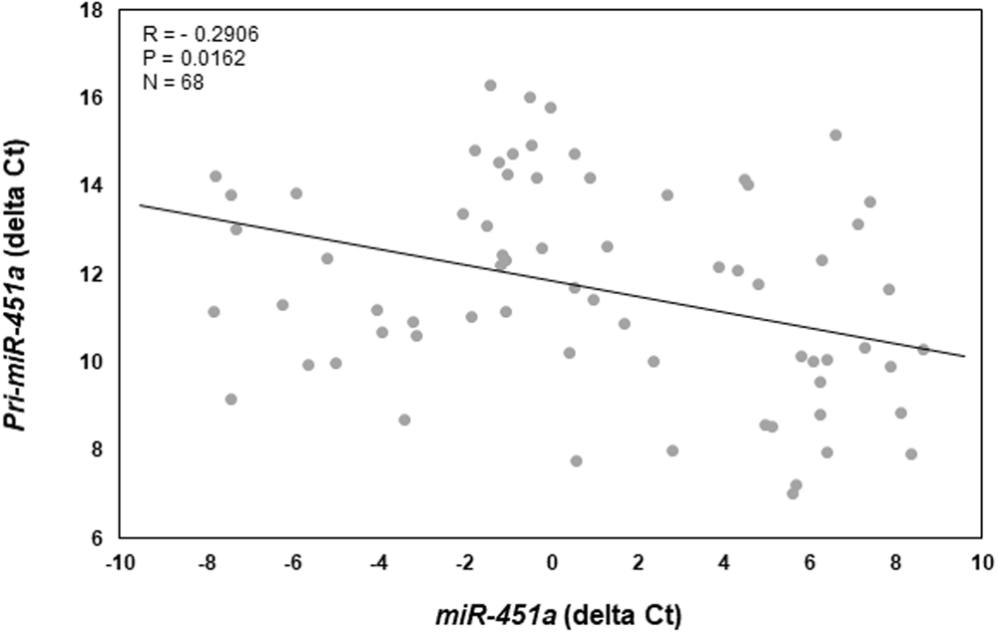
Correlation between mature *miR-451a* and pri-*miR-451a* in individual endometriotic lesions. Delta ct values (mature *miR-451a* or pri-*miR-451a* – U58) were plotted and Spearman’s correlation coefficient was calculated. There was a significant negative correlation in lesion expression between mature- and pri-*miR-451a*.

**Figure 5:**
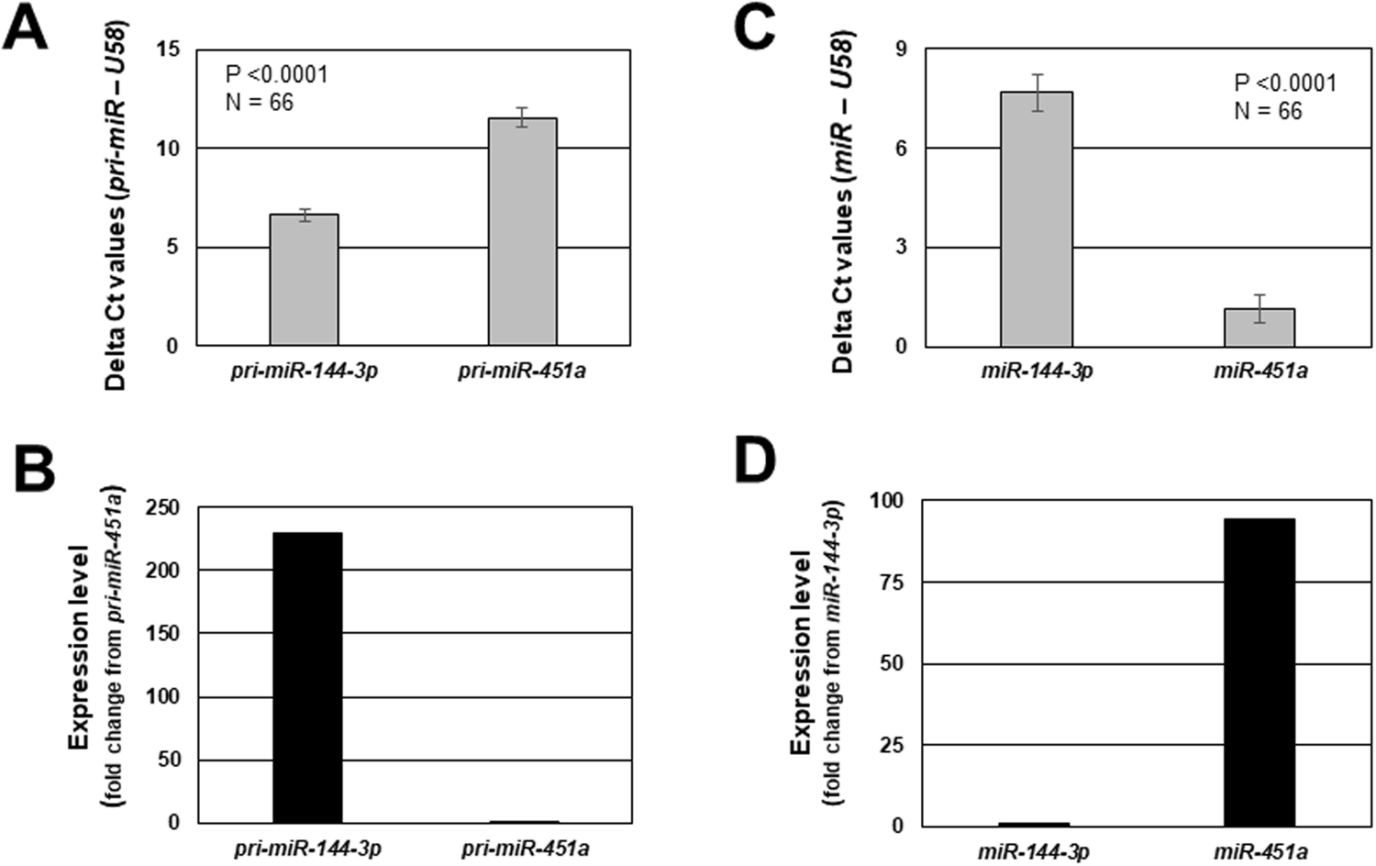
Comparison of average pri- and mature *miR-144-3p* and *miR-451a* expression in endometriotic lesion tissue. Pri-*miR-144-3p* and pri-*miR-451a* expression are presented as delta ct values (**A**) and fold change from pri-*miR-451a* (**B**) while mature *miR-144-3p* and *miR-451a* are presented as delta ct values (**C**) and fold change from *miR-144-3p* (**D**). These data are presented to emphasize the greater level of pri-*miR-144-3p* expression compared pri-*miR-451a*, but the greater expression of mature *miR-451a* despite the low levels of lesion pri-*miR-451a* expression.

Similarly, both pri-*miR-451a* (Fig. 3A) and mature *miR-451a* (Fig. 3B) expression was significantly greater in endometriotic lesion tissue compared to matched eutopic endometrium. Much like *miR-144-3p*, there was a great deal of variation within and among lesions with respect to expression levels of both pri-*miR-451a* and mature *miR-451a* (Fig. 3C). In contrast to *miR-144-3p*, we detected a significant, negative correlation (R = −0.2906; P < 0.05) between pri-*miR-451a* expression and that of mature *miR-451a* expression (Fig. 4). In comparing pri-forms of both *miR-144-3p* and *miR-451a* (Fig. 5A,B), we found that pri-*miR-144-3* was approximately more than 225-fold more abundant in endometriotic lesion tissue compared to pri-*miR-451a*. In contrast, mature *miR-451a* was approximately 90-fold more abundant in lesion tissue compare to mature *miR-144-3p* (Fig. 5C,D).

### Origin of mature miR-144-3p and miR-451a in endometriotic cells and lesions

**Figure 6:**
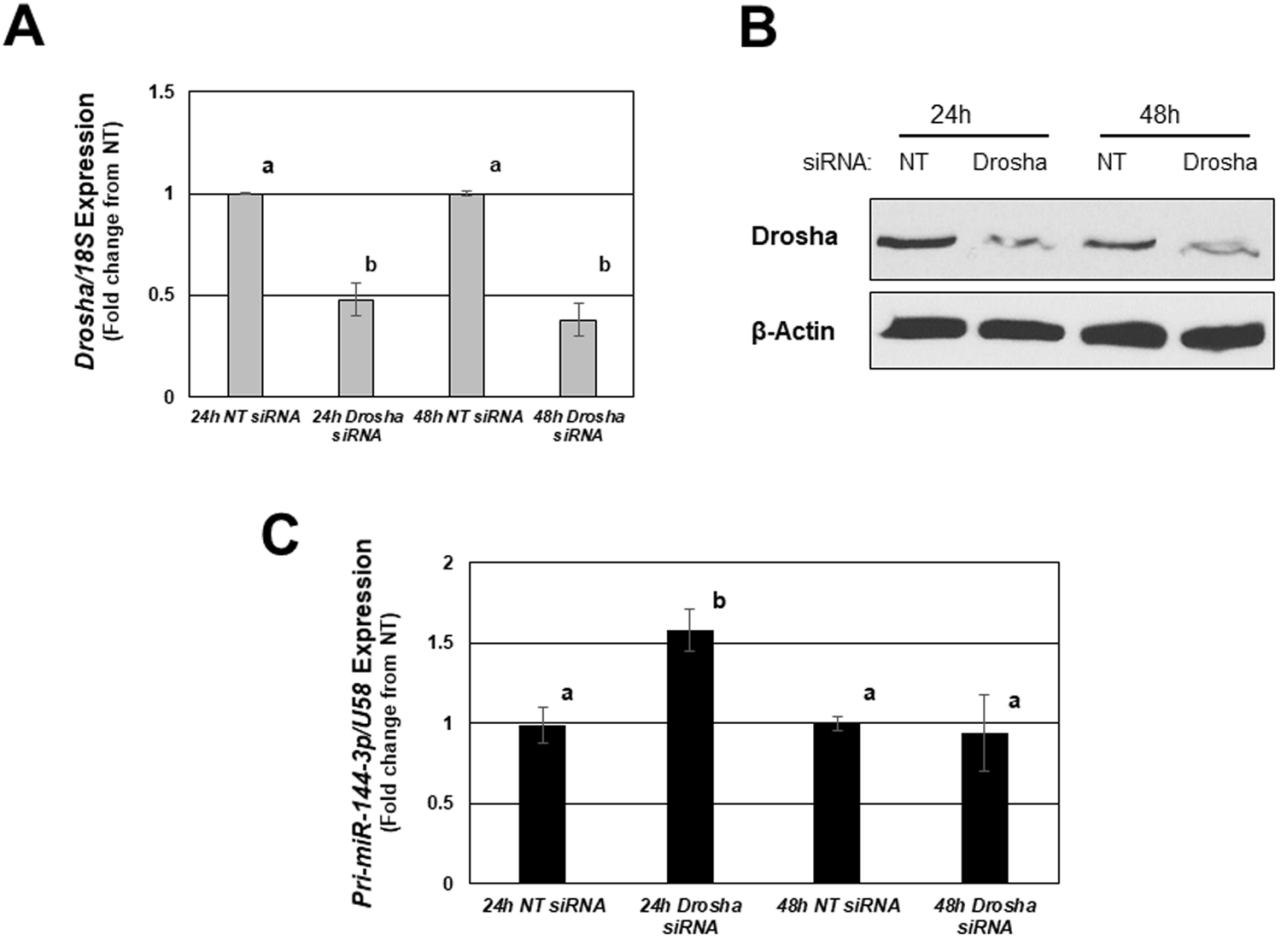
Inhibition of the miRNA processing enzyme, DROSHA, enriches endometriotic lesion expression of pri-*miR-144-3p* but not pri-*miR-451a*. The endometriotic epithelial cell line, 12Z was transfected with either a non-targeting siRNA (NT; negative control) or DROSHA siRNA. Twenty-four and forty-eight hours after transfection, DROSHA mRNA (**A**) and protein (**B**) expression were determined as was pri-*miR-144-3p* (**C**) and pri-*miR-451a* (data not shown). *Pri-miR-451a* was not detected in any of the 12Z cell samples by qRT-PCR. Different letters indicate statistical significance (P < 0.01) among the means by one-way ANOVA followed by Bonferroni post-hoc analysis (planned comparisons).

To begin to examine the mechanisms for the disparity between miRNA levels of expression, we transfected endometriotic epithelial 12Z cells with siRNA to DROSHA. As depicted in Fig. 6, transfection of 12Z cells with DROSHA siRNA, but not NT siRNA, resulted in significant reduction in transcript (Fig. 6A) and protein levels (Fig. 6B) at both 24- and 48-hours post transfection. This reduction in DROSHA expression was associated with a significant enrichment (approximate 55% increase) in pri-*miR-144-3p* expression suggesting that active processing of *miR-144-3p* from the genome occurred in 12Z cells. In contrast, knockdown of DROSHA had no effect on levels of pri-*miR-451a* at either 24 or 48-hours post transfection (data not shown).

**Figure 7:**
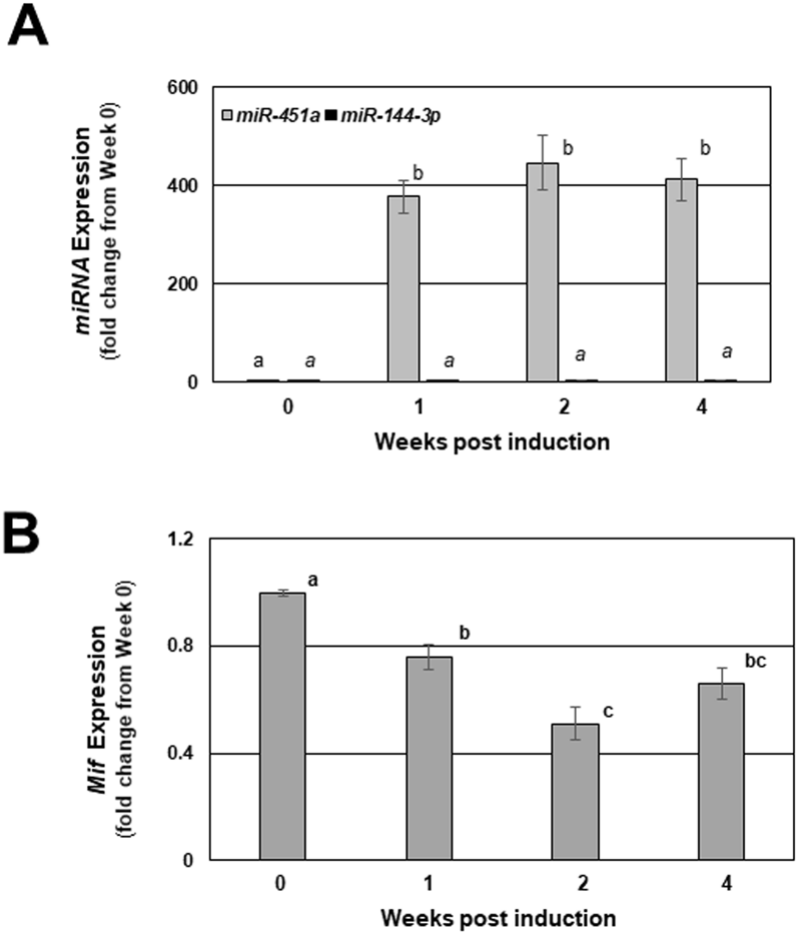
Mature *miR-144-3p*, *miR-451a*, and *Mif* transcript expression in endometriotic-like lesions from mice with experimentally induced endometriosis. Endometriosis was induced in wild-type mice using donor tissue from *miR-144*-*3p*/*miR-451a* null mice as described under “Materials and Methods” and mice were sacrificed at 1, 2 and 4 weeks post-induction (week 0 = time of induction). Endometriotic lesion-like structures were removed from each mouse, carefully minimizing underlying peritoneum contamination and total RNA was isolated. Murine *miR-144-3p* and *miR-451a* (**A**) were evaluated by qRT-PCR. *miR-451a* confirmed target transcript *Mif* was also evaluated by qRT-PCR in the same lesion tissues at each of the time points (**B**). Data are displayed as the mean ± SEM. Different letters indicate statistically significant differences among the means as determined by one-way ANOVA followed by post-hoc analysis (N = 6 per group; P < 0.05). Time 0 represents assessments made on tissue fragments (eventual lesions) that were harvested from donors but not transferred to recipient mice and served as the baseline for expression of the indicated miRNAs and their indicated targets.

These *in vitro* studies suggested that *miR-144-3p* may be transcribed from the genome, while the inability of DROSHA inhibition to enrich pri-*miR-451a* might suggest rapid processing of the pri-form of this miRNA or that lesion content may be derived from exogenous sources. Considering that *miR-451a* is a major miRNA found in the circulation and erythrocytes and that there is a rich vascularization of endometriotic lesion tissue, we examined the contribution of exogenous *miR-144-3p* and *miR-451a* to total lesion expression of these miRNAs. Figure 7 depicts expression levels of mature *miR-144-3p* and *miR-451a* in endometrial fragments from *miR-144-3p*/*miR-451a* deficient donor mice which were used to establish endometriotic-like lesions in our experimental animal model. As expected, endometrial tissue from *miR-144*/*miR-451* deficient mice did not express either miRNA. Assessment of *miR-144-3p* and *miR-451a* expression in this same *miR-144-3p*/*miR-451a* deficient tissue after various timepoints post-induction revealed a significant increase in *miR-451a*, but not *miR-144-3p* expression (Fig. 7A). To further confirm that the elevated expression of lesion *miR-451a* elicited a biological effect on lesion tissue, we evaluated *Mif* mRNA expression in this same mouse tissue. Associated with increased *miR-451a* expression was a significant reduction in the *miR-451a* target *Mif* (Fig. 7B). This observation is similar to that reported previously by us in human endometriotic lesion tissue^23^ and also validates our experimental mouse model as a model which can replicate aspects of the human disease.

## Discussion

This is the first study to examine expression of the *miR-144-3p/miR-451a* cluster in human endometrium and endometriotic lesion tissue as well as explore mechanisms for its expression using a novel animal model. Endometriosis is a complex disease in which inflammation is proposed to play a central role in its pathophysiology. Both members of the cluster (*miR-144-3p* and *miR-451a*) have been respectively proposed to target^21,22^ inflammatory factors associated with the pathogenesis of endometriosis including PTGS2/COX2^26–28^, TNF-α^29,30^, IL-1β^29,30^, IL-6^30,31^, MIF^23^, and YWHAZ^24^, but the majority of emphasis has been placed upon *miR-451a*. The present findings suggest that *miR-144-3p* is expressed in both eutopic and ectopic endometriotic lesion tissue, but a role for this miRNA in the pathophysiology of endometriosis has yet to be examined. *miR-144-3p* has been reported to regulate mediators of inflammation such as TNF-α, IL-6 and IL-1β^32,33^ as well as PTGS2/COX2^34^. Thus, it may be of interest to further evaluate the regulation of these inflammatory mediators by *miR-144-3p* in the context of endometriosis pathophysiology.

We observed that expression of both *miR-451a* and *miR-144-3p* was heterogeneous among lesions, even among similar types (red peritoneal lesions). This heterogeneity of expression cannot be attributed to enrichment/reduction of epithelium or stroma among lesions as we have examined cytokeratin (epithelial cell marker) and vimentin (stromal cell marker) expression in these samples (unpublished observation in this study as well as in a previous study^23^). Presentation of data from individual lesions (Figs 1 and 3) emphasizes the necessity to evaluate patterns of expression as opposed to simply reporting overall fold-change (increase or decrease from eutopic endometrium). An emerging “working” hypothesis of our research program is that as early endometriotic lesions (red lesions) progress towards becoming less active, *miR-451a* levels increase and functionally lead to reduced survival of the lesion. We are confident that the heterogeneity in the level of *miR-451a* expression represents physiological changes within the lesion and is not attributed to differences in lesion content of epithelium. This statement is based upon our observations that the levels of the epithelial markers, cytokerin-18 and cyokertin-19 do not correlate with *miR-451a* lesion content^23^.

Within the endometrium and endometriotic lesion tissue, *miR-451a* localizes predominantly to glandular epithelium^24^. Using the endometriotic epithelial cell line, 12Z as a model, we detected low levels of both the pri- and mature forms of *miR-451a* and relative levels of the former could not be enriched by knockdown of DROSHA expression. Although transcribed from the same locus, processing of *miR-144-3p* and *miR-451a* to the mature forms undergoing differential processing with *miR-451a* processing occurring via DICER independent mechanisms^35,36^. However, the initial step for both pri-miRNAs relies on DROSHA activity. As such, we postulated that reduction of DROSHA expression would result in an enrichment of *pri-miR-144-3p* and *pri-miR-451a*. While we did observe such an enrichment for *miR-144-3p* in 12Z cells in which DROSHA expression was reduced by siRNA, we did not observe this for *miR-451a*. These data were interpreted to suggest that *miR-451a* may be derived from sources other than the cellular genome of endometriotic lesion tissue. This concept was supported by low levels of 12Z cell expression of *miR-451a*. This postulate was also supported by our observation that expression of *pri-miR-451a* was exceptionally low in human endometriotic lesion tissue, while expression levels of mature *miR-451a* were rather robust.

Using our novel mouse model in which wild-type mice (which expressed both *miR-144-3p* and *miR-451a*) harbored lesion tissue derived from *miR-144-3p/miR-451a* deficient donor mice, we confirmed that “lesion” content consists of exogenous sources of *miR-451a*, as the gene for this miRNA cannot be expressed by this tissue and therefore must be derived from the host tissue/environment. We do not believe that *miR-451a*, although expressed by peritoneal mesothelial cells (unpublished observation), contributed to the lesion content of this miRNA. We base this statement on two facts. First, in our mouse model, endometriotic lesion tissue does not penetrate the underlying peritoneum and can be easily separate from that tissue. Thus, if there was any contribution from this tissue, it would be minimal. Second, as the levels of *miR-451a* (and MIF) changed over the time course of this study, the amount of potentially contaminating underlying peritoneum would not change, which supports the notion that the underlying peritoneum was not a major contributor to the level or pattern of *miR-451a* expression. Based upon these observations, we interpreted these results to suggest that endometriotic lesion *miR-144-3p* content may be derived from the cellular genome. In contrast, we believe that a significant proportion of *miR-451a* content may be derived from exogenous sources such as infiltrating erythrocytes and/or exosome crosstalk between cells within the circulation and endometriotic lesion.

Further proof of principal for a role of *miR-451a* in not only the pathophysiology of endometriosis, but perhaps also the therapeutic utility of *miR-451a* may be evaluated in non-human primate models. Joshi and colleagues^24^ demonstrated that *miR-451a* was mis-expressed in endometriotic lesion tissue compared to eutopic endometrium but only evaluating a single time point. Time course studies post induction may offer insight as to whether or not a similar pattern of expression might occur in this model as was observed in our mouse model.

While the current study has many strengths, we are aware that it also has limitations. First, we focused on red peritoneal lesions and ovarian endometriomas and did not assess additional lesions such as deep-infiltrating lesions. It would be of interest in future studies to evaluate if deep-infiltrating lesions also express a similar pattern of *miR-144-3p/miR-451a* expression as well as further explore their pathophysiology. Second, we limited the study to only those women who did not receive hormonal therapy within a minimal 3-month period prior to surgery. It may be of interest to see of hormonal therapies have any impact on *miR-144-3p/miR-451a* expression in cases of failed treatment to learn more about the regulation and role of these miRNAs in lesion survival and symptoms of the disease. These shortcomings, as well as the potential therapeutic utility of *miR-451a* in endometriosis treatment may be evaluated in future studies. In summary, we report for the first-time elevated expression of the *miR-144-3/miR-451a* cluster in human endometriotic lesion tissue which correlates with survival status of the lesion and that *miR-451a*, but not *miR-144-3p*, may be derived from infiltrating or invading sources.

## Materials and Methods

### Ethical approval and study subjects

The study was approved by the institutional review boards of both the University of Kansas Medical Center and Cleveland Clinic and all experiments were performed in accordance with relevant guidelines and regulations of those institutions. Written informed consent was obtained prior to surgical removal of endometriotic lesion tissue and endometrial biopsies. Following similar approaches to those previously reported^37^, samples were obtained from a total of 48 women (N = 48) between the ages of 21 and 45. Women with endometriosis who presented with pelvic pain due to failed previous endometriosis treatment and were undergoing surgical removal of endometriotic lesion tissue for failed endometriosis treatment were enrolled. A total of 48 subjects were enrolled (N = 18 in the proliferative stage of the menstrual cycle, N = 30 in the secretory stage of the menstrual cycle and included women with stage I/II (N = 15) and stage III/IV (N = 33) endometriosis (Table 1). No subjects had taken GnRH analogs or hormonal therapy within 3 months prior to surgery. A total of 48 endometrial biopsies (eutopic endometrium) and 68 matched (same patient) endometriotic lesions (N = 58 red peritoneal lesions and N = 10 ovarian endometriomas) were collected at the time of surgical removal of endometriosis of all types including deep infiltrating lesions (which were not used for research purposes). All specimens were collected by the same surgeon (TF, RF) at Cleveland Clinic and (KS) at the University of Kansas Medical Center with emphasis on minimizing sample contamination from underlying/surrounding non-endometriotic lesion tissue. To do so, endometriotic lesions were excised and sent to pathology for confirmation of endometriosis, which was defined as the presence of endometrial glands and stroma. Tissue was excised using sharp scissors with no energy. During the excision the underlying tissue was separated from the lesion tissue. Research samples from Cleveland Clinic were immediately snap-frozen, stored at −80 °C and then shipped to the University of Kansas Medical Center, while samples obtained from the University of Kansas Medical Center were transported on ice to the research laboratory where they were snap-frozen upon arrival. Stage of the menstrual cycle was determined from the patient’s medical records with day 1 defined as the onset of menses.

**Table 1:** Patient Demographics.

Study group/age range	Diagnosis/stage of menstrual cycle	Lesion type (N)^1^
**Endometriosis** (**N** = **48**) 21–45 years of age		**Lesion type** (**N** = **68**)
	Stage I/II endometriosis (N = 18)	
	Proliferative (N = 6)	P (7), O (2)
	Secretory (N = 12)	P (15), O (2)
	Stage III/IV endometriosis (N = 30)	
	Proliferative (N = 9)	P (12), O (2)
	Secretory (N = 21)	P (24), O (4)

^1^N indicates the number of each lesion type within group. Abbreviations: P = peritoneal biopsy; red lesion, O = ovarian endometrioma.

Samples were subjected to RNA extraction followed by quantitative real-time (qRT)-PCR analysis as described below. As no difference in any of the pri- or mature forms of the miRNAs assessed was noted among stages of the menstrual cycle, stages of endometriosis, type of lesion (peritoneal or ovarian endometrioma) or influenced by medications, data were collapsed and analyzed as ectopic versus eutopic tissue for each endpoint.

### RNA isolation and qRT-PCR of miRNAs and mRNAs

RNA was isolated from tissue using Trizol (Life Technologies) at 1.0 mL of Trizol/100 mg of tissue following the protocols provided by the manufacturer. Total RNA (1 µg in 20 µl) was reverse transcribed using reverse transcription (RT) kits (Applied Biosystems; Foster City, CA) following the manufacturer’s protocol specific for each pri- and mature miRNA. Total RNA (250 ng in 5 µl) was reverse transcribed using RT kits (Applied Biosystems) following the manufacturer’s protocol with the following modifications. Briefly, miRNAs were reverse transcribed in a single reaction using 2 µl of each miRNA specific 5X RT primers. Resulting material was then used for independent qRT-PCR for each miRNA. To normalize for starting material, a reverse snRNA U58 was included in the miRNA RT reactions and qRT-PCR of U58 was performed using validated primers from Applied Biosystems. mRNA was reversed transcribed using 1 µg of total RNA using reverse transcription (RT) kits (Applied Biosystems; Foster City, CA) following the manufacturer’s protocol. Primers for DROSHA were designed using Primer-Blast and synthesized by Integrated DNA Technology (IDT, Coralville, IA). Sequences for the human *DROSHA* (NM_013325) primers were: forward, 5′-CTGGCAAGGGCATTCACAT-3′ and reverse, 5′-TGATTGTGGCCTAGGGTCAGA-3′. DROSHA expression levels were normalized to 18S rRNA using primers from Applied Biosystems). All qRT-PCR reactions were completed on a QuantStudio7 Flex Real-Time PCR System (Applied Biosystems). All samples were run in triplicate and the average value used in subsequent calculations. The 2-delta-delta CT method was used to calculate the fold-change values among samples as previously described by our group^23,37^. qRT-PCR intra- and inter-assay coefficients of variation were both less than 5%.

### Cell culture of endometriotic epithelial 12Z cells and siRNA transfection

The endometriotic epithelial cell line, 12Z (originally described by Zeitvogel *et al*.^38^) was obtained from Dr. Linda Griffith (Massachusetts Institute of Technology, Cambridge, MA). Cell culture was conducted following the general approach as previously described^34^. Briefly, cells were cultured in phenol red-free Dulbecco’s Minimum essential medium (DMEM)/Ham’s F12 (Fisher Scientific, Pittsburgh, PA) + 10% charcoal stripped FBS (Atlanta Biologicals, Atlanta, GA) + Pen-Strep (Life Technologies, Carlsbad, CA) in T75 flasks and seeded at 1 × 10^6^ cells/ml of media until approximately 90% confluency. Cells were then passed and plated in 6-well plates at a density of 1 × 10^5^ cells/ml in DMEM/Ham’s F12 media lacking FBS and Pen-Strep. The next day, cells were transfected as described below for each specific experiment.

To assess the impact of DROSHA on processing of *pri-miR-144-3p* and *pri-miR-451a*, 12Z cells were transfected with DROSHA siRNA (Dharmacon RNAi Technologies, Lafayette, CO) or a non-targeting (NT) siRNA (50 nM final concentration for each). Briefly, 12Z cells were cultured in phenol red-free DMEM:F12 supplemented with 10% charcoal-stripped FBS, penicillin, and streptomycin. Cells were transfected at 50% confluency using Lipofecateamine-2000 transfection agent according to recommendations of the manufacturer (Applied Biosystems) using siRNAs specific for human *DROSHA* transcripts (Dharmacon RNAi Technologies, Lafayette, CO) or control, non-targeting sequences (Dharmacon). Twenty-four to forty-eight hours after transfection, DROSHA levels were assessed by Western blotting and qRT-PCR.

### Western blotting

Western blot analysis was conducted as previously reported^37^. Briefly, total protein was extracted from cell culture samples using RIPA buffer (1X RIPA, Catalog #9806, Cell Signaling Technologies, Danvers, MA). Protein concentration in each sample was determined using the Bio-Rad Protein Assay (Bio-Rad Laboratories, Richmond, CA). The same amount of protein (30 µg) was subjected to 12% Bis (2-hydroxyethyl) amino-tris (hydroxymethyl) methane (w/v) gel electrophoresis and electroblotted onto nitrocellulose membranes (GE Healthcare, Pittsburgh, PA). Rabbit anti-DROSHA (ab183732; 1:10,000; Abcam Inc., Cambridge, MA) and goat anti-rabbit secondary antibody (1:5000; GE Healthcare) were used. Stripping and re-probing for beta-actin (ab8227; Abcam; 1:10,000 dilution) was conducted to normalize DROSHA protein expression levels. Immunodetection was carried out using an enhanced chemiluminescence (ECL) kit (Thermo Scientific, Waltham, MA).

### Mouse model of endometriosis

All animal experiments were conducted at the University of Kansas Medical Center under the guidance of Dr. Nothnick following the relevant guidelines and regulations. Experimental procedures incorporating animals were approved by the University of Kansas Medical Center Institutional Animal Care and Use Committee (IACUC). Experimental endometriosis was induced as previously described with modifications^39^. Briefly, 22- to 24-day old C57BL/6 female mice deficient for *miR-451a*/*miR-144-3p* were injected s.c. with pregnant mare serum gonadotropin (PMSG; 2 IU; Sigma Chemical Company, St. Louis, MO) to stimulate endogenous estrogen production and subsequent estrogenic response within the uterus. Uteri were then harvested from these donors 42–44 h after PMSG injection. Uterine stroma and epithelium (endometrium) was separated from myometrium with the aid of a dissecting microscope. Endometrial tissue (which contained stromal as well as glandular and luminal epithelium) was cut into 10 fragments of equal size (1 mm^3^). Uterine fragments were suspended in 0.4 mL of sterile saline containing 12.5% v/v Matrigel (Corning Life Sciences, Corning, NY). Recipient mice (2- to 4-month old wild-type C57BL/6 immuno-competent, reproductively intact females, which express *miR-451a*/*miR-144-3p*) were anesthetized with ketamine/xylazine and an antibiotic ointment was placed over the corneas to avoid corneal abrasions. The area over the right rib cage was prepared for surgery and a small incision (approximately 0.5 cm) was made exposing the peritoneal cavity. Tissue fragments were injected into the peritoneal cavity through the incision and the incision was then closed with wound clips. Carprofen analgesic was given post-operatively at the conclusion of the surgery and again 24 h later. Mice were then sacrificed at indicated time post endometriosis induction. In this model, established endometriotic lesion will not express *miR-451a*/*miR-144-3p* from the genome and expression of *miR-451a*/*miR-144-3p* would be from infiltrating cells into the lesions.

### Statistical analysis

Pri- and mature miRNA levels were first separately assessed within stage of endometriosis (stage I/II vs. stage III/IV in endometriosis subjects) and among stage of menstrual cycle. As no significant differences among pri- or mature miRNAs expression could be attributed to stage of endometriosis, type of endometriotic lesion (peritoneal or ovarian endometrioma) or stage of menstrual cycle, data were pooled and analyzed as eutopic endometrial tissue compared to endometriotic lesion tissue. All data were first assessed for normal (Gaussian) distribution. Data which displayed normalcy of distribution were analyzed by paired t-tests or one-way ANOVA where appropriate with Bonferroni post-hoc analysis. Data which failed to display normality of distribution were analyzed by non-parametric tests as specified below. Differences in the delta Ct values among subject groups or among time points (baboon sera across different times post endometriosis induction) were analyzed using Kruskal-Wallis test followed by post-hoc analysis using Dunn’s multiple comparison test. To examine the correlation among pri- and mature forms of each miRNA, Spearman correlation coefficients were calculated. All analysis was conducted using GraphPad Prism6 (GraphPad Software, La Jolla, CA). Significance was set at P < 0.05 for all analyses.

## References

[1] Giudice LC, Kao LC (2004). Endometriosis. Lancet..

[2] Laganà AS (2017). Anxiety and depression in patients with endometriosis: impact and management challenges. Int. J. Womens Health..

[3] Vitale SG, La Rosa VL, Rapisarda AMC, Laganà AS (2017). Impact of endometriosis on quality of life and psychological well-being. J. Psychosom. Obstet. Gynecol..

[4] Vitale SG, Petrosino B, La Rosa VL, Rapisarda AM, Laganà AS (2016). A systematic review of the association between psychiatric disturbances and endometriosis. J. Obstet. Gynecol. Can..

[5] Halme J, Hammond MG, Hulka JF, Raj SG, Talbert LM (1984). Retrograde menstruation in healthy women and in patients with endometriosis. Obstet. Gynecol..

[6] Liu DT, Hitchcock A (1986). Endometriosis: its association with retrograde menstruation, dysmenorrhoea and tubal pathology. Br. J. Obstet. Gynaecol..

[7] Zhang T, De Carolis C, Man GCW, Wang CC (2018). The link between immunity, autoimmunity and endometriosis: a literature update. Autoimmune Rev..

[8] Bałkowiec M, Maksym RB, Włodarski PK (2018). The bimodal role of matrix metalloproteinases and their inhibitors in etiology and pathogenesis of endometriosis (Review). Mol. Med. Rep..

[9] Nothnick W, Alali Z (2016). Recent advances in the understanding of endometriosis: the role of inflammatory mediators in disease pathogenesis and treatment. F1000Res..

[10] Patel BG (2018). Pathogenesis of endometriosis: Interaction between endocrine and inflammatory pathways. Best Pract. Res. Clin. Obstet. Gynaecol..

[11] Laganà AS, Salmeri FM, Vitale SG, Triolo O, Götte M (2018). Stem cell trafficking during endometriosis: may epigenetics play a pivotal role?. Reprod. Sci..

[12] Vetvicka V (2016). Regulation of apoptotic pathways during endometriosis: from the molecular basis to the future perspectives. Arch. Gynecol. Obstet..

[13] Panir K, Schjenken JE, Robertson SA, Hull ML (2018). Non-coding RNAs in endometriosis: a narrative review. Hum. Reprod. Update..

[14] Bartel DP (2004). MicroRNAs: genomics, biogenesis, mechanism, and function. Cell..

[15] Erson AE, Petty EM (2008). MicroRNAs in development and disease. Clin. Genet..

[16] Ohlsson Teague EM (2009). MicroRNA-regulated pathways associated with endometriosis. Mol. Endocrinol..

[17] Filigheddu Nicoletta, Gregnanin Ilaria, Porporato Paolo E., Surico Daniela, Perego Beatrice, Galli Licia, Patrignani Claudia, Graziani Andrea, Surico Nicola (2010). Differential Expression of MicroRNAs between Eutopic and Ectopic Endometrium in Ovarian Endometriosis. Journal of Biomedicine and Biotechnology.

[18] Hawkins SM (2011). Functional microRNA involved in endometriosis. Mol. Endocrinol..

[19] Braza-Boils A (2014). MicroRNA expression profile in endometriosis: its relation to angiogenesis and fibrinolytic factors. Hum. Reprod..

[20] Haikalis ME, Wessels JM, Leyland NA, Agarwal SK, Foster WG (2018). MicroRNA expression pattern differs depending on endometriosis lesion type. Biol. Reprod..

[21] Agarwal V, Bell GW, Nam JW, Bartel DP (2015). Predicting effective microRNA target sites in mammalian mRNAs. Elife..

[22] Tokar T (2018). mirDIP 4.1-integrative database of human microRNA target predictions. Nucleic Acids Res..

[23] Graham A, Falcone T, Nothnick WB (2015). The expression of microRNA-451 in human endometriotic lesions is inversely related to that of macrophage migration inhibitory factor (MIF) and regulates MIF expression and modulation of epithelial cell survival. Hum. Reprod..

[24] Joshi NR (2015). Altered expression of microRNA-451 in eutopic endometrium of baboons (Papio Anubis) with endometriosis. Hum. Reprod..

[25] Dore LC (2008). GATA-1 regulated microRNA locus essential for erythropoiesis. Proc. Natl. Acad. Sci. USA..

[26] Cobellis L (2004). The treatment with a COX-2 specific inhibitor is effective in the management of pain related to endometriosis. Eur. J. Obstet. Gynecol. Reprod. Biol..

[27] Olivares C (2008). Effects of a selective cyclooxygenase-2 inhibitor on endometrial epithelial cells from patients with endometriosis. Hum. Reprod..

[28] Santulli P (2014). Hormonal therapy deregulates prostaglandin-endoperoxidase synthase 2 (PTGS2) expression in endometriotic tissue. J. Clin. Endocrinol. Metab..

[29] Keenan JA, Chen TT, Chadwell NL, Torry DS, Caudle MR (1995). IL-1beta, TNF-alpha, and IL-2 in the peritoneal fluid and macrophage-conditioned media of women with endometriosis. Am. J. Reprod. Immunol..

[30] Bergqvist A (2000). Production of interleukins 1beta, 6 and 8 and tumor necrosis factor alpha in separated and cultured endometrial and endometriotic stromal and epithelial cells. Gynecol. Obstet. Invest..

[31] Tsudo T (2000). Altered gene expression and secretion of interleukin-6 in stromal cells derived from endometriotic tissue. Fertil. Steril..

[32] Li D (2015). Down-regulation of miR-144 elecits proinflammatory cytokine production by targeting toll-like receptor 2 in nonalcoholic steatohepatitis of high-fat-diet-induced metabolic syndrome E3 rats. Mol. Cell Endocrinol..

[33] Li RD (2018). MicroRNA-144 suppresses the expression of cytokines through targeting RANKL in the matured immune cells. Cytokine..

[34] Li H (2016). miR-144 and targets, c-fos and cyclooxygenase-2 (COX2), modulate synthesis of PGE2 in the amnion during pregnancy and labor. Sci. Rep..

[35] Cheloufi S, Dos Santos CO, Chong MM, Hannon GJ (2010). A dicer-independent miRNA biogenesis pathway that requires Ago catalysis. Nature..

[36] Cifuentes D (2010). A novel miRNA processing pathway independent of Dicer requires Argonaute 2 catalytic activity. Science..

[37] Nothnick WB (2018). Macrophage migration inhibitory factor receptor, CD74, is overexpressed in human and baboon (Panio Anubis) endometriotic lesions and modulates endometriotic epithelial cell survival and interleukin-8 expression. Reprod. Sci..

[38] Zeitvogel A, Baumann R, Starzinski-Powitz A (2001). Identification of an invasive, N-cadherin-expressing epithelial cell type in endometriosis using a new cell culture model. Am. J. Pathol..

[39] Nothnick WB, Graham A, Holbert J, Weiss MJ (2014). miR-451 deficiency is associated with altered endometrial fibrinogen alpha chain expression and reduced endometriotic implant establishment in an experimental mouse model. PLoS. One..

